# Draft Genome Sequence of Potassium-Dependent Alkaliphilic *Bacillus* sp. Strain TS-2, Isolated from a Jumping Spider

**DOI:** 10.1128/genomeA.00458-14

**Published:** 2014-05-22

**Authors:** Shun Fujinami, Kiyoko Takeda, Takefumi Onodera, Katsuya Satoh, Motohiko Sano, Issay Narumi, Masahiro Ito

**Affiliations:** a Bio-Nano Electronics Research Centre, Toyo University, Kawagoe, Saitama, Japan; b United Graduate School of Agricultural Science, Tokyo University of Agriculture and Technology, Fuchu, Tokyo, Japan; c Cooperative Research Center of Life Sciences, Kobe Gakuin University, Kobe, Hyogo, Japan; d Ion Beam Mutagenesis Research Group, Medical and Biotechnological Application Unit, Quantum Beam Science Center, Japan Atomic Energy Agency, Takasaki, Gunma, Japan; e Graduate School of Life Sciences, Toyo University, Itakura-machi, Gunma, Japan; f Faculty of Life Sciences, Toyo University, Itakura-machi, Gunma, Japan

## Abstract

The potassium-dependent alkaliphilic *Bacillus* sp. strain TS-2 was isolated from the mashed extract of a jumping spider, and its draft genome sequence was obtained. Comparative genomic analysis with a previously sequenced sodium-dependent alkaliphilic *Bacillus* species may reveal potassium-dependent alkaline adaptation mechanisms.

## GENOME ANNOUNCEMENT

The typical alkaliphilic *Bacillus* species (e.g., *Bacillus pseudofirmus* OF4 and *Bacillus halodurans* C-125) have a sodium-dependent alkaline adaptation mechanism and show sodium-dependent growth and motility (1, 2). However, some potassium-requiring alkaliphiles have also been reported (3, 4), suggesting the existence of a potassium-dependent alkaline adaptation mechanism. Thus, we have isolated some potassium-dependent alkaliphiles and reported one of their draft genome sequences (5). Here, we report the draft genome sequence of another potassium-dependent alkaliphile, *Bacillus* sp. strain TS-2. This bacterium was isolated from the mashed extract of a jumping spider and appeared to be most closely related to *Bacillus pseudalcaliphilus*, based on 16S rRNA gene sequence identity.

The draft genome sequence of *Bacillus* sp. strain TS-2 was 4,360,646 bp in total length, comprised 58 large contigs (>500 bp), and was obtained using the Roche GS Junior and assembled by GS *de novo* assembler v. 2.7. Automatic annotation was performed using the Microbial Genome Annotation Pipeline (6), which predicted a total of 4,085 protein-coding genes. The names of products of the predicted protein-coding genes were revised manually. tRNA detection was performed using the ARAGORN software (7), which predicted a total of 73 tRNAs.

Typical alkaliphilic *Bacillus* species have an *atpE* gene that encodes an F_1_F_o_-H^+^-ATPase *c* subunit, and the amino acid residue that is critical for H^+^ selectivity under alkaline pH conditions has already been identified (8). The annotation of the draft genome sequence shows that *Bacillus* sp. strain TS-2 has an *atpE* gene. The multiple sequence alignment of the ATPase *c* subunit suggested that the *atpE* gene of this bacterium encodes an H^+^-ATPase *c* subunit that is proposed to function under alkaline pH conditions.

Typical alkaliphilic *Bacillus* species also have a *motPS* gene that encodes an Na^+^-dependent flagellar motor stator protein, and the amino acid residue that is critical for coupling ion selectivity has been identified (2, 4). In the draft genome sequence of *Bacillus* sp. strain TS-2, a *motPS* gene was annotated. The multiple sequence alignment of flagellar motor stator proteins suggested that the *motPS* gene of this bacterium encodes an Na^+^-dependent flagellar motor stator protein. It would be interesting to analyze the function of this protein in the future.

In the draft genome sequence of *Bacillus* sp. strain TS-2, two sets of *mrp* genes, which encode multisubunit secondary cation/proton antiporter-3 family proteins, were annotated. The Mrp complex acts as an Na^+^/H^+^ antiporter in typical alkaliphilic *Bacillus* species and plays a critical role in the sodium-dependent alkaline adaptation mechanism (1, 2). In draft genome sequence analysis of sodium-independent alkaliphilic *Microbacterium* sp. strain TS-1, three sets of *mrp* genes were reported (5). Therefore, multiple sets of *mrp* genes might also support the use of various cations as a coupling ion of H^+^ influx for alkaline adaptation.

Future genome analysis of other sodium-independent alkaliphiles may reveal common features of novel alkaline adaptation mechanisms.

### Nucleotide sequence accession numbers.

The draft genome sequence of *Bacillus* sp. strain TS-2 was deposited at DDBJ/EMBL/Genbank under the accession number BAWL00000000. The version described in this paper is the first version, BAWL01000000.
